# Plastid encoded RNA polymerase activity and expression of photosynthesis genes required for embryo and seed development in *Arabidopsis*

**DOI:** 10.3389/fpls.2014.00385

**Published:** 2014-08-12

**Authors:** Dmitry Kremnev, Åsa Strand

**Affiliations:** Umeå Plant Science Centre, Department of Plant Physiology, Umeå UniversityUmeå, Sweden

**Keywords:** chloroplast, PEP, NEP, embryo development, photosynthesis, PRIN2, CSP41b

## Abstract

Chloroplast biogenesis and function is essential for proper plant embryo and seed development but the molecular mechanisms underlying the role of plastids during embryogenesis are poorly understood. Expression of plastid encoded genes is dependent on two different transcription machineries; a plastid-encoded bacterial-type RNA polymerase (PEP) and a nuclear-encoded phage-type RNA polymerase (NEP), which recognize distinct types of promoters. However, the division of labor between PEP and NEP during plastid development and in mature chloroplasts is unclear. We show here that PLASTID REDOX INSENSITIVE 2 (PRIN2) and CHLOROPLAST STEM-LOOP BINDING PROTEIN 41 kDa (CSP41b), two proteins identified in plastid nucleoid preparations, are essential for proper plant embryo development. Using Co-IP assays and native PAGE we have shown a direct physical interaction between PRIN2 and CSP41b. Moreover, PRIN2 and CSP41b form a distinct protein complex *in vitro* that binds DNA. The *prin2.2* and *csp41b-2* single mutants displayed pale phenotypes, abnormal chloroplasts with reduced transcript levels of photosynthesis genes and defects in embryo development. The respective *csp41b-2prin2.2* homo/heterozygote double mutants produced abnormal white colored ovules and shrunken seeds. Thus, the *csp41b-2prin2.2* double mutant is embryo lethal. *In silico* analysis of available array data showed that a large number of genes traditionally classified as PEP dependent genes are transcribed during early embryo development from the pre-globular stage to the mature-green-stage. Taken together, our results suggest that PEP activity and consequently the switch from NEP to PEP activity, is essential during embryo development and that the PRIN2-CSP41b DNA binding protein complex possibly is important for full PEP activity during this process.

## INTRODUCTION

The chloroplasts house the photosynthetic light reactions where sunlight is converted into chemical energy. Plastids are also the location of a number of vital metabolic pathways, including primary carbon metabolism and the biosynthesis of fatty acids, amino acids, and tetrapyrroles. Chloroplast function is required throughout the life cycle of the plant and compromised activity can result in embryo lethality. Abortion of developing embryos is known to occur when amino acid, nucleotide or fatty acid biosynthesis is impaired, or when import of chloroplast proteins and translation are disrupted (McElver et al., 2001; Tzafrir et al., 2003; Hsu et al., 2010; Bryant et al., 2011). In contrast, disrupting components of the photosynthetic apparatus leads to reduced pigmentation and changed physiology rather than embryo lethality (Tzafrir et al., 2003; Bryant et al., 2011). Chloroplasts are detected as early as at the globular stage of the embryo (Tejos et al., 2010) and transcript profiling during embryo development showed a significant increase in the expression of nuclear genes encoding components involved in energy production, carbon fixation and photosynthesis already from the globular embryogenic stage (Spencer et al., 2007; Le et al., 2010; Belmonte et al., 2013). Furthermore, it has been indicated that chloroplasts can provide the embryo with energy and O_2_ required for biosynthesis and respiration (Rolletschek et al., 2003). Additionally, it was shown that *Brassica* embryos were able to fix CO_2_ and contributing to embryo growth rate and biomass (Goffman et al., 2005).

Chloroplasts, like mitochondria, evolved from free-living prokaryotic organisms that entered the eukaryotic cell through endosymbiosis. The gradual conversion from endosymbiont to organelle during the course of evolution has clearly been accompanied by a dramatic reduction in genome size as the chloroplasts lost most of their genes to the nucleus. The genes remaining in the chloroplast genome are related to photosynthesis or encode components of the plastid gene expression machinery (Wakasugi et al., 2001; Martin et al., 2002). The chloroplast genes of higher plants are transcribed by at least two types of RNA polymerases; the nuclear encoded plastid RNA polymerase (NEP), a T3-T7 bacteriophage type that predominantly mediates the transcription of the house keeping genes (Hedtke et al., 1997, 2000; Puthiyaveetil et al., 2010). The other type, plastid encoded RNA polymerase (PEP) is a bacterial-type multi-subunit enzyme that predominantly mediates the transcription of photosynthesis-related genes (Allison et al., 1996; De Santis-MacIossek et al., 1999). Most chloroplast genes can be transcribed by both polymerases but they utilize different promoter elements (Hajdukiewicz et al., 1997; Pfannschmidt and Liere, 2005). The PEP enzyme recognizes the -10 and -35 *cis*-elements, similar to those found in bacterial promoters whereas the NEP enzyme recognizes the YRTA-motif, which can also be found upstream of several genes with PEP promoters indicating that these genes can be transcribed by both polymerases (Pfannschmidt and Liere, 2005). Transcription of the plastid encoded photosynthesis genes during chloroplast development and the activation of the photosynthetic reactions are accompanied by a switch from NEP to PEP activity (Hanaoka et al., 2005). However, the mechanisms underlying this change in major RNA polymerase activity and the division of labor between NEP and PEP in the chloroplast are unknown (Zhelyazkova et al., 2012).

A large number of proteins have been shown to be associated with the PEP complex and the components associated with PEP changes in response to developmental signals and changes in the environment (Pfalz and Pfannschmidt, 2013). The variation in the composition of the PEP complex suggests that regulation of plastid gene expression is both complex and sophisticated. PLASTID REDOX INSENSITIVE 2 (PRIN2) is a novel plant protein localized to the plastid nucleoids and plastid transcriptome analyses demonstrated that PRIN2 is required for full expression of genes transcribed by PEP (Kindgren et al., 2012). The role of the PEP associated proteins is unclear and whether these proteins contribute to the regulation of plastid gene expression by environmental and developmental cues remains to be determined. In order to shed light on the complex regulation of PEP activity and to understand the function of PRIN2 we pursued an assay to identify interacting protein partners of PRIN2. We report here a direct interaction between PRIN2 and CHLOROPLAST STEM-LOOP BINDING PROTEIN 41 kDa (CSP41b) using three different biochemical methods. CSP41b was described as an RNA binding protein identified in nucleoid preparation and was attributed numerous plastid functions, for example CSP41b was suggested to stimulate both transcription and translation in the chloroplast (Pfannschmidt et al., 2000; Yamaguchi et al., 2003; Suzuki et al., 2004; Hassidim et al., 2007; Bollenbach et al., 2009). We also demonstrate that PRIN2 and CSP41b form a distinct DNA binding protein complex *in vitro* and that the *csp41b-2prin2.2* double mutant is embryo lethal. Taken together, our results suggest that PEP activity and consequently the switch from NEP to PEP activity, is essential also during embryo development and that the PRIN2-CSP41b protein complex potentially is important for full PEP activity during this process.

## MATERIALS AND METHODS

### PLANT MATERIAL AND GROWTH CONDITIONS

Seedlings of Arabidopsis thaliana were grown on phytoagar plates containing 1 × Murashige and Skoog salt mixture supplemented with vitamins (Duchefa) and 2% sucrose. The T-DNA insertion lines: prin2.2 (GK-772D07-024643) and csp41b-2 (SALK_021748), were obtained from the European Arabidopsis Stock Centre. All genotypes are in Colombia ecotype. Seedlings and rosette plants were grown on soil at 23°C (16 h light 150 μmol photons m^-2^ s^-1^) and 18°C (8 h dark).

### MORPHOLOGICAL ANALYSIS

Embryo isolation was done according to (Perry and Wang, 2003). Briefly, The siliques at the desired stage of DAP were dissected under a stereo microscope and the seeds were collected. Embryos were gently extruded from the seed coat by applying pressure on a glass plate covering them. Embryo/seed coat mixture was loaded a 25% Percoll gradient in isolation buffer (10 mM potassium phosphate, pH 7.0, 50 mM NaCl, 0.1 M sucrose) and centrifuged for 10 min at 800 g. The pellet was subjected to another round of purification using the 25% Percoll gradient. The pellet was re-suspended in isolation buffer and used for subsequent analysis. For transmission electron microscopy (TEM) pictures, 3-weeks-old plants grown on soil and the isolated seed embryos were prepared according to (Barajas-Lopez Jde et al., 2013).

### RNA ISOLATION, cDNA SYNTHESIS AND REAL-TIME PCR

Total RNA was isolated using Plant RNA Mini Kit (EZNA) and genomic DNA contamination was removed by DNase treatment (Fermentas). cDNA was synthesized using the iScript cDNA kit (Bio-Rad) according to the manufacturer’s instructions and 10 × diluted. Real-Time PCR was performed using iQSYBR Green Supermix (Bio-Rad), with a final volume of 10 μL. The PCR amplification was done using two-step protocol using the CFX96 Real-Time system (C1000 Thermal Cycler; Bio-Rad). All experiments were performed with three biological and three technical replicates, the relative gene expression was normalized to the expression of RCE1 (At4g36800) and PP2AA3 (At1g13320). Data analysis was done by CFX manager (Bio-Rad) and LinRegPCR software.

### EXPRESSION AND PURIFICATION OF RECOMBINANT PROTEINS

The coding sequences of PRIN2 and CSP41b were amplified with PCR. The PCR products were cloned using the NcoI–AccI sites into pET_His1a vector. BL21 *Escherichia coli* cells were transformed with the expression constructs and induced for 6 h with 1 mM IPTG. Overexpressed proteins were affinity purified on Ni^2+^-NTA agarose resin (Qiagen). The pET-His1a expression vector was kindly provided by Günter Stier, Umeå University, Sweden.

### EMSA

The 197 bp probe containing -196 to +1 PsaA promoter region was PCR amplified and labeled at the 3′-end with biotin-14-dCTP using biotin labeling kit (Invitrogen) according to the manufacturer’s instructions. DNA–protein interactions were performed in 25 mcL reactions containing following reagents: 2.5 mcL of × 10 binding buffer (100 mM Tris HCl, 250 mM KCl, and 10 mM DTT), 1 mcg poly dIdC (Sigma–Aldrich), 2,5% glycerol, 0.05% Triton X-100, 5 mM MgCl2, 10 mM EDTA. The reaction mixture was incubated with DNA and protein at room temperature for 30min and was run on 6% native TBE-PAGE in x0,5 TBE buffer at 100 V. DNA was transferred to nylon + membrane (Amersham) and was UV cross-linked to the membrane, incubated with Streptavidin-HRP and detected by Chemoluminescence Nucleic Acid Detection Module (Pierce) according to manufacturer’s instructions (Shaikhali et al., 2012a,b).

### Co-IP

To identify PRIN2 interacting partners, a 35S promoter linked to the full length PRIN2 coding sequence was cloned into the pGWB16_myc expression construct. Col-0 plants were then transformed with 35S_pGWB16myc_PRIN2 using the floral-dip method (Clough and Bent, 1998). Two weeks old stable transformants overexpressing PRIN2 were used for chloroplast isolation in a two-step 50–25% Percoll gradient as described previously (Aronsson and Jarvis, 2002). Chloroplast proteins were extracted and incubated with 3 mg of anti-cMYC monoclonal antibody (Bio-Site) bound to the protein G-coated magnetic beads (Dynabeads Protein G Immunoprecipitation, Invitrogen) for 1 h at 4°C. All the washing steps were performed at 4°C according to the manufacturer’s instructions. Immunoprecipitated PRIN2 protein complexes were eluted in SDS loading buffer containing 100 mM β-mercaptoethanol and proteins were separated on 6–12% SDS PAGE. Non-transformed Col-0 plants were used as negative control. For direct protein Co-IP assay, full length PRIN2 and CSP41b coding sequences were cloned into *NcoI–NcoI* sites of pRT104_3myc and into *SacI–NotI* sites of pRT104_3HA vector, respectively. Protoplasts obtained from *Arabidopsis Ler-0* cell culture were transformed with pRT104_PRIN2_myc and pRT104_CSP41b_HA constructs as described previously by (Doelling and Pikaard, 1993).

### ISOLATION OF THYLAKOID COMPLEXES AND BLUE NATIVE PAGE

Chloroplasts were isolated on a two-step 50–25% Percoll gradient from 4 weeks old rosette plants grown in short day as described previously (Aronsson and Jarvis, 2002). Thylakoid membranes were then purified (Hall et al., 2011) and protein complexes were solubilized in BN-solubilization buffer (30 mM HEPES, pH 7.4; 150 mM potassium acetate; 10% glycerole, 4% digitonin (SIGMA); 1% *b*-Dodecylmaltoside (SIGMA) for 40 min, 4°C. DM and digitonin in a mixture was shown to be suitable for better preservation of megacomplexes and at the same time good for solubilization (Järvi et al., 2011). 35 micro gram protein was loaded onto the 4–12% Bis-Tris Gel (NuPAGEH Novex 1.0 mm, Invitrogen) from each genotype.

## RESULTS

### PRIN2 AND CSP41B FORM A DISTINCT PROTEIN COMPLEX THAT BINDS DNA

**FIGURE 1:**
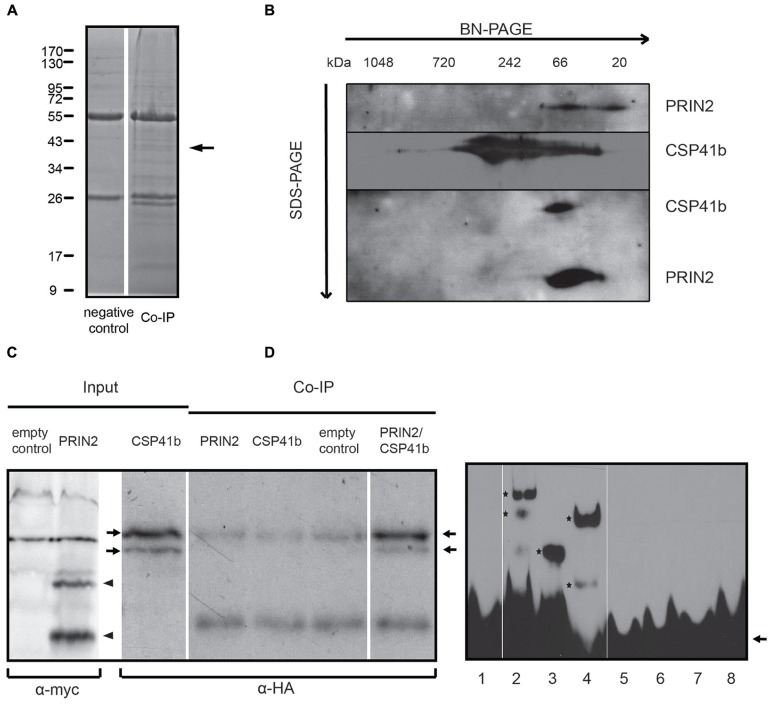
**PRIN2 interacts with CSP41b and forms a DNA-binding complex. (A)** Native PRIN2-cMyc protein complex from *Arabidopsis* chloroplasts was immunoprecipitated with anti-cMyc antibody and proteins were separated on the SDS-PAGE. Non-transformed *Col-0* chloroplasts were used as a negative control. Protein bands were cut from the gel and analyzed by mass spectrometry. An arrow indicates the position of the band where CSP41b was identified. **(B)** PRIN2 interaction with CSP41b detected by 2D Native PAGE/SDS PAGE gel. Recombinant PRIN2, CSP41b and 1:1 mixture of both proteins were incubated in the binding buffer and run on the 4–12% Native Bis-Tris Gel. Individual stripes were then cut and loaded on the 12% SDS PAGE gel. Proteins were detected with anti-His peroxidase.** (C)** Interaction between PRIN2 and CSP41b *in vivo* Co-IP assay. Full length PRIN2-cMyc and CSP41b-HA fusion proteins were expressed in *Arabidopsis* protoplasts and Co-IP was performed using anti-cMyc antibody linked to protein G-covered magnetic beads. Immunoblotted samples from input and Co-IP probes were detected with either anti-cMyc chicken IgY fraction and rabbit HRP-linked anti-chicken IgY (H+L) or with anti-HA peroxidase. Arrowheads indicate PRIN2 protein and arrows show CSP41b protein bands detected by anti-HA antibody. **(D)** DNA binding of PRIN2 and CSP41b and their heteromerization in EMSA assay. Signal from *psaA*-198 bp biotin labeled probe was detected by chemoluminescence nucleic acid detection module. 3 μg of each purified protein was used in every reaction, the DNA/protein molar ratio was 1:100. DNA/protein complexes are marked with asterisks, free DNA probe with an arrow. Competition was done with unlabeled *psaA*-198 bp probe with 50-fold excess over the labeled probe. 3 μg of BSA was used as a negative control. (1) free probe, (2) probe + PRIN2, (3) probe + CSP41b, (4) probe + PRIN2 + CSP41b, (5) probe + PRIN2 + unlabeled probe, (6) probe + CSP41b + unlabeled probe, (7) probe + PRIN2 + CSP41b + unlabeled probe, (8) probe + BSA.

To understand the function of PRIN2 we wanted to identify proteins interacting with PRIN2 *in vivo*. To achieve this a co-IP approach was used. Full length PRIN2 protein fused to a cMyc-tag was expressed in *Arabidopsis* plants, intact chloroplasts were isolated and PRIN2-containing protein complexes were precipitated with anti-cMyc antibody, proteins were separated on SDS PAGE and distinct bands identified using mass spectrometry (**Figure 1A**). The Co-IP experiment was performed twice and as many as 17 bands absent from the negative controls were in total cut from the gels. Most of the identified proteins were only found in one experiment and most likely represented unspecific interactions. However, one protein, CSP41b was identified in both experiments and several peptides with significant scores corresponding to CSP41b were identified in each sample (Table S1 in Supplementary Material). CSP41b is a conserved chloroplast protein and the *csb41b-2* mutant displayed impaired chloroplast transcription and plant development (Pfannschmidt et al., 2000; Suzuki et al., 2004; Bollenbach et al., 2009; Qi et al., 2012). The phenotype of the *csb41b-2* mutant was very similar to the phenotype of the *prin2* mutant and it is possible that CSP41b and PRIN2 are involved in the same process. Thus, the identified interaction between PRIN2 and CSP41b was chosen for further analysis.

First, to really confirm the interaction between CSP41b and PRIN2 purified recombinant proteins were separated on 2D electrophoresis (**Figure 1B**; Table S2). PRIN2 forms protein complexes ranging from 20–66 kDa while CSP41b is present in high molecular weight complexes ranging from 40–700 kDa. When PRIN2 and CSP41 are incubated together, PRIN2 seems to break the high molecular weight complexes of CSP41b and form a distinct protein complex with CSP41b suggesting heteromerization. To further confirm *in vivo* that PRIN2 and CSP41b directly interact with each other we used a third method by transiently expressing the full length proteins PRIN2 and CSP41b fused to cMyc- and HA-tags, respectively, in *Arabidopsis* protoplasts. Two bands of approximately 50 kDa in size correspond to CSP41b in the CSP41b-HA transformed protoplasts and these could be cytoplasmic/transite peptide-processed forms of the protein and/or proteolytic fragment of the protein. The PRIN2 protein exists in monomeric, dimeric and oligomeric forms (unpublished data) and migrates under these conditions as two bands of approximately 20 and 40 kDa, respectively, most likely corresponding to monomer and dimer. CSP41b was detected in the Co-IP fraction confirming interaction with PRIN2 (**Figure 1C**). Thus, using three independent methods CSP41b and PRIN2 were shown to directly interact.

Both PRIN2 and CSP41b were suggested to regulate transcription of PEP dependent chloroplast genes (Bollenbach et al., 2009; Kindgren et al., 2012). To investigate if PRIN2 and CSP41b interact upon DNA binding *in vitro*, the proteins were incubated with a 197 bp DNA probe containing -196 to +1 region of the *psaA* promoter from *Arabidopsis*. In the electrophoretic mobility shift assay (EMSA) both PRIN2 and CSP41b bound the labeled probe, PRIN2 formed at least two distinct complexes with DNA, while CSP41b formed only one DNA-protein complex (**Figure 1D**). PRIN2 and CSP41b are about 15 and 40 kDa, respectively, and the difference in the migration of the protein/DNA complexes of PRIN2 and CSP41b suggests that PRIN2 forms higher molecular weight oligomeric complexes and/or binds to several regions of the DNA probe. When PRIN2 and CSP41b were incubated together with DNA, a new band, intermediate in size to what was observed for the individual proteins, was detected that most likely corresponded to a *psaA*197-PRIN2/CSP41b heteromeric complex. The competition reactions with unlabeled *psaA*-198 bp confirm the DNA binding capacity of PRIN2 and CSP41b. A similar pattern of DNA-binding could also be observed when using the *psbA*-198 bp fragment as a probe, where PRIN2 and CSP41b proteins form heteromeric protein/DNA complexes (Supplementary Figure S1). Thus, both PRIN2 and CSP41b are able to interact with DNA in the EMSA assay and the formation of the heteromeric PRIN2/CSP41b complex also occurs upon DNA binding *in vitro*.

### THE *prin2.2* AND *csp41b-2* MUTANTS SHOW DISTINCT PHENOTYPES AND IMPAIRED EXPRESSION OF CHLOROPLAST ENCODED GENES

**FIGURE 2:**
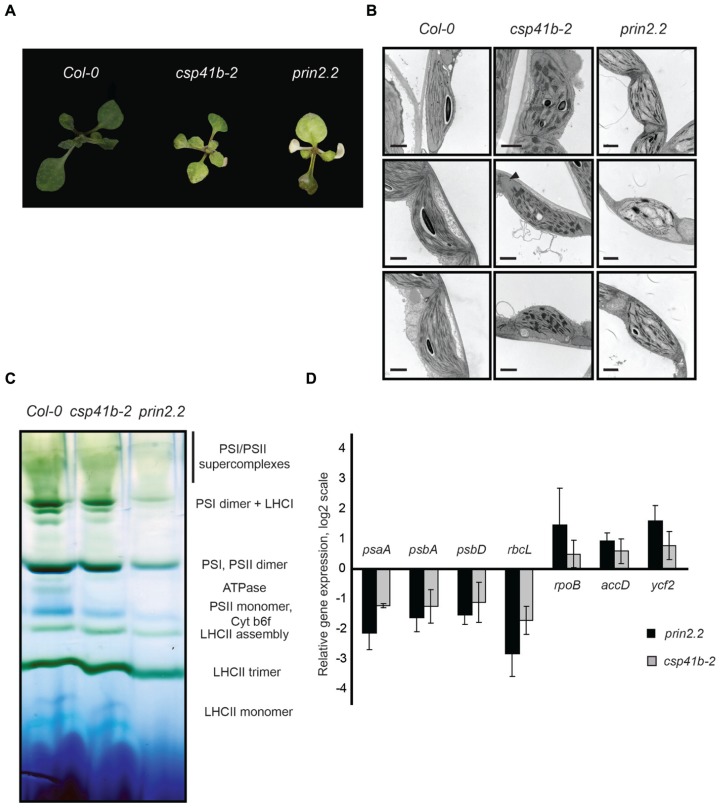
**The* prin2* and *csp41b-2* show severe plant phenotypes.** Plants were grown on soil under long day conditions (16 h light/8 h darkness) to characterize *prin2.2* and *csp41b-2* mutants **(A)** Representative images of plants grown on soil for 3 weeks. **(B)** Representative TEM images of chloroplasts from the Col-0, *csp41b-2,* and *prin2.2* rosette plants. Bars represent 1 μm **(C)** Blue Native PAGE of thylakoid membrane complexes isolated from Col-0,* csp41b-2,* and *prin2.2* chloroplasts **(D)** Relative expression levels of chloroplast encoded genes in *prin2.2* and *csp41b-2* mutant plants. Expression levels were compared to the respective Col-0 samples and calculated using Ubiquitin-protein ligase (At4g36800) as a reference gene. Data represents the mean from three independent biological replicates.

The phenotypes of the *prin2.2* and *csp41b-2* mutants are similar to mutants of defined components of the PEP complex (De Santis-MacIossek et al., 1999; Krause et al., 2000; Legen et al., 2002; Pfalz et al., 2006). As has been demonstrated before *prin2.2* and *csp41b-2* showed a clear reduction in growth rate and pale leaves compared to wild type (**Figure 2A**). The *prin2.2* plants have impaired chloroplast structure with reduced thylakoid membranes and grana stacks (**Figure 2B**). In contrast, the *csp41b-2* mutant showed an increased number of thylakoid membranes organized in grana stacks (**Figure 2B**). Moreover, large areas of the chloroplasts (depicted with an arrowhead) were devoid of any thylakoid membranes in the *csp41b-2* mutant. Correlated with aberrant chloroplast structure is the observed defect in the organization of the photosynthetic complexes in the *prin2.2* and *csp41b-2* mutants. The mutants showed reduced amounts of PSI, PSII, ATPase, and antenna complexes compared to wild type (**Figure 2C**). The defect in photosynthetic complex stoichiometry and organization was especially strong for the *prin2.2* mutant.

Expression analyses were performed for genes categorized as genes primarily transcribed by PEP and NEP, respectively. The *psaA*, *psbA, psbD,* and *rbcL* genes belong to class I genes transcribed by the PEP polymerase. Consistent with previous studies (Bollenbach et al., 2009; Kindgren et al., 2012; Qi et al., 2012), both *prin2.2* and *csp41b-2* mutants showed decreased *psaA*, *psbA, psbD,* and *rbcL* expression levels compared to wild type. In contrast, the expression levels of the NEP genes, *accD, rpoB, ycf2* were elevated compared to wild type (**Figure 2D**). The chloroplast transcriptional machinery that primarily utilizes PEP polymerase is clearly impaired in the *prin2.2* and *csp41b-2* mutants and when the two mutants were compared side by side the effect was stronger in the *prin2.2* mutant.

### THE *prin2.2* AND *csp41b-2* MUTANTS SHOW DEFECTS IN EMBRYO DEVELOPMENT AND THE *csp41b-2 prin2.2* DOUBLE MUTANT IS EMBRYO LETHAL

**FIGURE 3:**
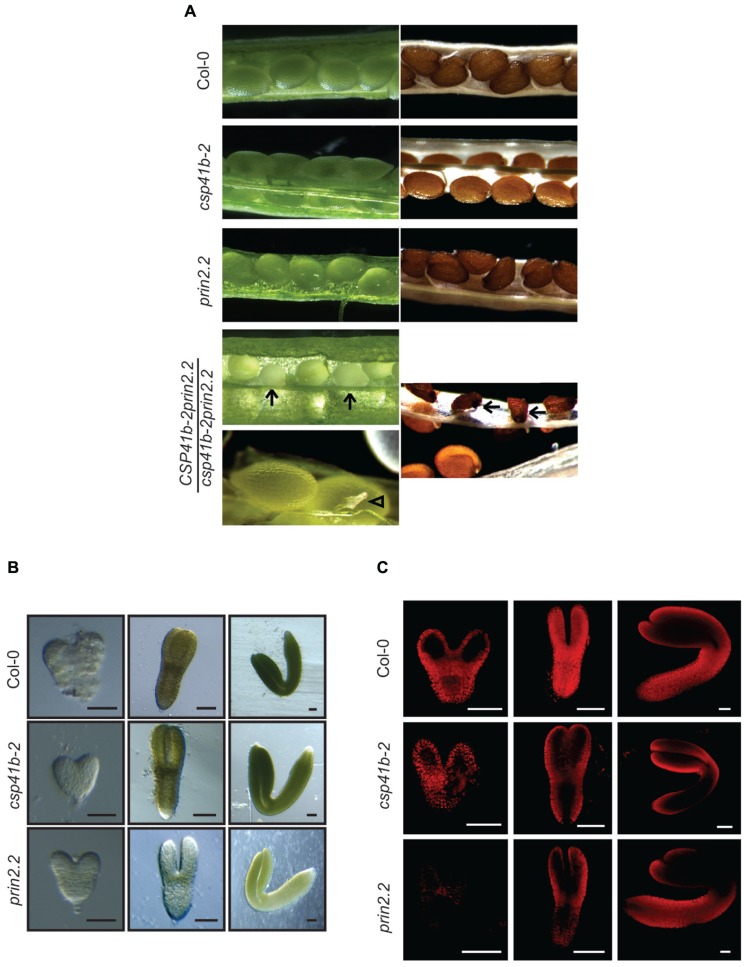
**The *csp41b-2prin2.2/csp41b-2prin2.2* double mutant is lethal. (A)** Siliques from self-fertilized *CSP41b-2prin2.2/csp41b-2prin2.2* double mutant. Arrows indicate aborted seeds and arrowheads show aborted ovules. **(B)** Embryo development of Col-0, *csp41b-2,* and *prin2.2*. Representative images are shown for each genotype. Bars represent 50 μm. **(C)** Chlorophyll autofluorescence in Col-0, *csp41b-2,* and *prin2.2* during specific stages of embryo development detected using confocal microscopy (Excitation wavelength 488 nm, Emission wavelength 687 nm). Representative images are shown for each genotype. Bars represent 50 μm.

In order to investigate the genetic interaction between PRIN2 and CSP41b we attempted to generate a *csp41b-2prin2.2* double mutant (Supplementary Figure S2). However, the *csp41b-2prin2.2* double mutant was embryo lethal and the *CSP41b-2prin2.2/csp41b-2prin2.2* mutant produced siliques where 18% (green:albino ovule = 240:51, Chi-square 8,67 for *p* < 0,05) of all ovules appeared opaque (**Figure 3A**, arrows). Those impaired ovules finally turned into shrunken, dark colored seeds unable to germinate on MS media. A few ovules were also aborted at very early developmental stages (**Figure 3A**, arrowheads). The fact that theoretical 3:1 segregation green/albino is not supported by our statistics could be explained by the observed range in the stage at which the embryo development is arrested. Given the embryo lethality of the double mutant we investigated if there were any effects during embryo development in the *csp41b-2* and *prin2.2* single mutants. The *csp41b-2* embryos were undistinguishable from the wild type at the heart stage (**Figure 3B**). However, at the linear cotyledon and mature green (MG) stages the *csp41b-2* embryos were not as uniformly green as the wild type embryos. As has been shown before wild type embryos displayed a specific pattern of chlorophyll autofluorescence during embryogenesis (Tejos et al., 2010). This pattern was significantly altered at the linear cotyledon stage (LC), where the chloroplast containing tissue was mostly localized to the epidermal layers, in the *csp41b-2* embryos (**Figure 3C**). Distribution of chloroplast containing tissue was even more altered in the *prin2.2* embryos (**Figure 3C**). Consistent with these findings are the light microscopy pictures demonstrating that the *prin2.2* embryos were paler than wild type embryos at all developmental stages (**Figure 3B**).

**FIGURE 4:**
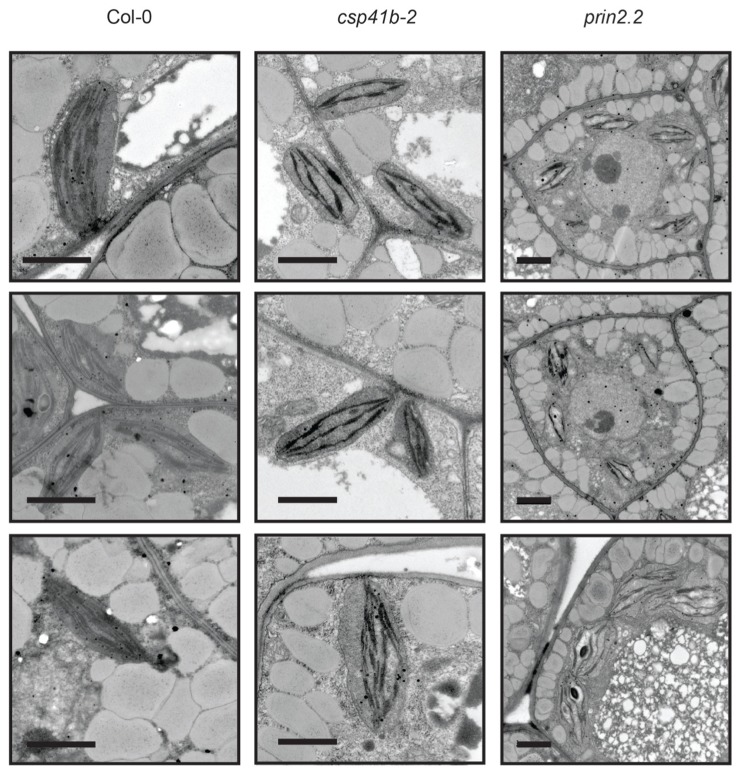
**The *csp41b-2* and* 2prin2.2* mutants display impaired chloroplast during embryo development.** Representative TEM images of chloroplasts from the Col-0, *csp41b-2,* and *prin2.2* mature green (MG) embryos. Bars represent 1 μm.

To investigate the potential role of PRIN2 and CSP41b during embryo development, we used TEM to examine morphological differences in the embryos at the MG stage. Plastids of wild type embryos develop normally and have numerous thylakoids organized in grana stacks indicating that the plastids can be photosynthetically functional at this stage of embryo development (**Figure 4**). However, *csp41b-2* chloroplasts developed less thylakoid membranes and fewer grana stacks and exhibit quite often chloroplasts with large areas completely devoid of membranes (**Figure 4**). This specific defect in chloroplast structure of *csp41b-2* was also observed for the chloroplasts from 3-week-old plants (**Figure 2B**). The *prin2.2* showed an even stronger impairment in chloroplast development, the *prin2-2* plastids showed numerous vesicles and few thylakoid membranes and grana stacks. Thylakoid membranes were also often mis-oriented (**Figure 4**). Similarly to what was observed for the *csp41b-2* mutant the chloroplasts from the embryos of *prin2.2* showed similar defects to what was also seen in the adult plants (**Figure 2B**). Taken together our results indicate that chloroplast development in the embryo is impaired in both *prin2.2* and *csp41b-2* single mutants and that the ovules are arrested at early developmental stages in the *csp41b*-2*prin2.2* double mutant.

### EXPRESSION OF PHOTOSYNTHESIS GENES ESSENTIAL DURING EMBRYO DEVELOPMENT

**FIGURE 5:**
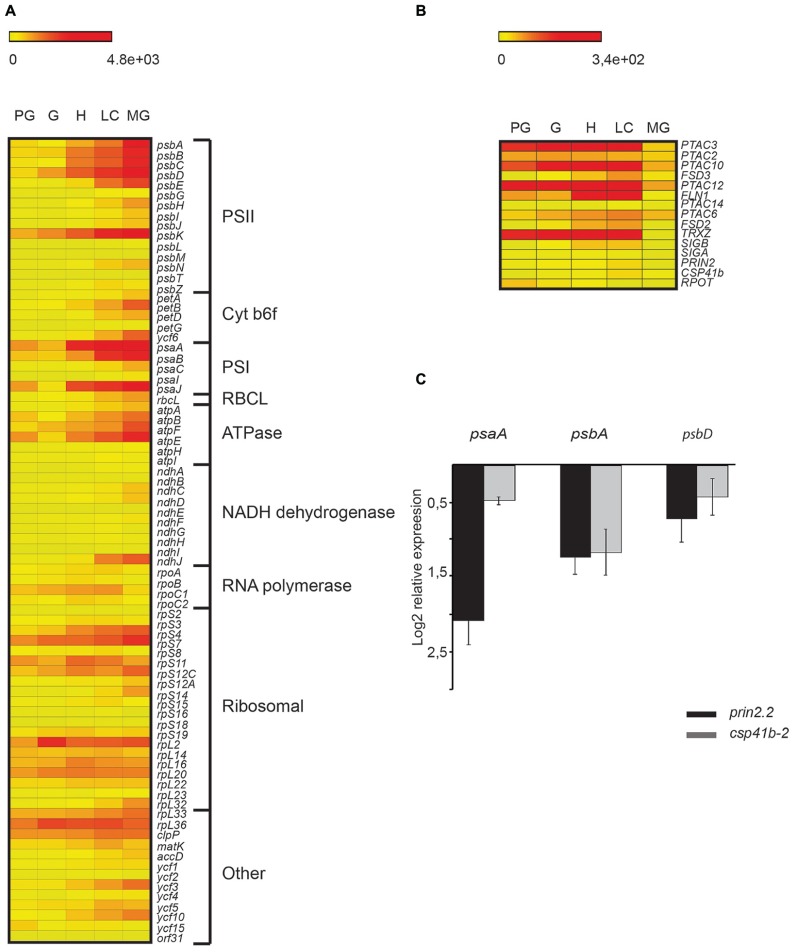
**Induction of photosynthetic gene expression during embryo development. (A)** Heatmap of plastid gene expression in Col-0 embryos at different developmental stages. Scale from 0 (yellow) to 4.8.e^3^ (red) represents the relative mean signal intensity for each probe **(B)** Heatmap of selected nuclear genes in wild type embryos at different developmental stages. Scale from 0 (yellow) to 3.4.e^2^ (red) represents the relative mean signal intensity for each probe. **(A)** and **(B)**: PG, preglobular stage; G, globular stage; H, heart stage; LC, linear cotyledon stage; MG, mature green embryo stage. **(C)** Expression levels of *psaA, psbA*, and* psbD* in *prin2.2* and *csp41b-2* MG embryos compared to the respective embryos of Col-0. Relative expression was calculated using *PP2AA3* (At1g13320) as a reference gene. Data represent means from three independent biological replicates.

We performed *in silico* analysis of available array data where gene activity was profiled genome-wide in every organ, tissue, and cell type of *Arabidopsis* seeds from fertilization through maturity (Belmonte et al., 2013). We specifically investigated the expression of plastid encoded genes from preglobular (PG) to MG embryo stage. Overall transcription of chloroplast-encoded photosynthesis associated genes was activated from globular to MG embryo stage (**Figure 5A**). The highest fold change in expression level was observed for the genes encoding PSI and PSII core subunits, ATPase, and the ribosomal subunits (**Figure 5A**). Thus, from the analysis of the plastid transcriptome it is clear that photosynthesis associated components are highly expressed during embryo development and that therefore PEP mediated transcription most likely is activated during this process. Expression of the nuclear encoded components associated with the PEP complex was demonstrated to increase from PG to LC (**Figure 5B**). Especially the genes encoding the PTAC proteins showed a very strong upregulation during embryo development. In addition, transcript levels of *TRXZ* were significantly up-regulated during the transition from PG to LC stage. Also transcription of *PRIN2* and *CSP41b* were observed during the LC stage of embryo development (**Figure 5B**).

The observed embryo phenotype in the *prin2.2* and *csp41b-2* mutants encouraged us to study expression of *psaA, psbA,* and *psbD* at the MG embryo stage. Expression of *psaA, psbA,* and *psbD* was strongly down regulated both in *prin2.2* and *csp41b-2* mutants compared to wild type (**Figure 5B**; Supplementary Figure S3). Thus, these results suggest that also under embryo development PRIN2 and CSP41b are important for proper transcription of PEP genes. Taken together, these results suggest that the PEP component of the chloroplast transcription machinery is active during embryo development and that its activity is essential for proper embryo and seed development.

## DISCUSSION

In leaf tissue, the initiation of chloroplast development in the light and the activation of the photosynthetic reactions are accompanied by repression of NEP activity and an increase of PEP-mediated plastid transcription (Hanaoka et al., 2005). However, the mechanisms underlying this change in major RNA polymerase activity and the division of labor between NEP and PEP in the chloroplast are unknown (Zhelyazkova et al., 2012). Expression of plastid-encoded photosynthetic components, thought to be mediated exclusively by PEP, was recently shown both in tobacco and barley to be driven by NEP in the absence of functional PEP, suggesting a less strict division of target genes between NEP and PEP (Legen et al., 2002; Lyubetsky et al., 2011; Zhelyazkova et al., 2012). NEP is particularly active in non-green tissues and in very young leaves (Emanuel et al., 2006). Similarly, the expression of the previously described NEP-dependent genes was distinctly higher in developing compared with mature chloroplasts (Zoschke et al., 2007), indicating that NEP is very active during the early stages of chloroplast biogenesis. However, we have demonstrated that PEP activity is essential during embryo development in *Arabidopsis*. During embryo development NEP was unable to compensate for impaired PEP activity in the *prin2.2* and *csp41b-2* mutants. Thus, the switch from NEP- to PEP-dependent transcription of the plastid-encoded genes is essential for embryo and seed development in *Arabidopsis*.

*In silico* analyses of available microarray data (Belmonte et al., 2013) showed a gradual increase in expression of genes encoding components involved in photosynthesis and energy production from the PG to the MG stage of embryo development (**Figure 5A**). During embryogenesis, expression of *RPOT* encoding the plastid NEP polymerase was induced and the maximum expression level was reached as early as at the PG and globular stages (G; **Figure 5B**). The *RPOT* expression then diminished already at the heart stage (H; **Figure 5B**). The NEP promoter is the only promoter in the *rpoBC* operon, and expression of *rpoA, rpoB,* and *rpoC1/C2* followed the induction of *RPOT*. The core components of PEP were transcribed from the globular stage but in contrast to *RPOT*, expression was maintained all through the different embryo developmental stages (**Figure 5A**). The expression profile of the nuclear encoded sigma factors, especially SIGB (SIG2) was similar to the profiles of *rpoA, rpoB,* and *rpoC1/C2* (**Figure 5B**). Correlated with the induction of the genes encoding the core components of PEP was a very strong induction of the plastid encoded photosynthesis genes. Expression of *psbA,B,C,D,E,* and *psaA,B,J*, for example, increased approximately 10-fold when the MG embryo was compared to the PG suggesting that PEP transcription is activated during this developmental transition. The strong induction of photosynthesis related mRNAs during the PG to MG transition was also shown in a study where three periods of seed formation was investigated (Allorent et al., 2013). The embryo lethality of the *csp41b-2prin2.2* double mutant further indicates that PEP driven transcription is required for effective transcription of photosynthesis genes to sustain the embryo with energy. It was also shown previously that genes associated with photosynthesis and carbon metabolism, are active in all embryo and endosperm sub-regions during early seed development (Belmonte et al., 2013) strongly suggesting there is a need for photosynthetic activity to contribute to embryo growth rate and biomass. Interestingly, at the MG embryo stage transcription of the nuclear encoded components of PEP was halted, suggesting that there is no need for further expression of chloroplast genes when the seeds enter the post-MG stage. In contrast, the chloroplast encoded photosynthesis genes showed a delayed response and maintained high expression also at the MG embryo stage. Possibly chloroplast transcription is subject to later anterograde regulation from the nucleus to repress expression.

True to its cyanobacterial origin, the core subunits of PEP are homologous to the cyanobacterial RNAP components (Martin et al., 2002). However, PEP also requires additional nuclear-encoded factors for its function (Pfannschmidt et al., 2000; Pfalz et al., 2006). As many as 40–60 proteins appear to be present in the TAC from chloroplasts. From Arabidopsis and mustard TACs 35 components were identified and 18 of those components, called pTACs, were novel proteins (Pfalz et al., 2006). In addition, TAC and sRNAP preparations from pro-plastids, chloroplasts and etioplasts have different protein compositions, suggesting a multifaceted regulation of PEP activity (Reiss and Link, 1985; Pfannschmidt and Link, 1994; Suck et al., 1996). Very high expression levels were observed for genes encoding the pTAC components PTAC3, PTAC10, PTAC12, FLN1, PTAC6, and TRXZ during the globular (G) to LC stages. Expression of *PRIN2* and *CSP41B* was also detected during the LC stage. The expression of all these additional PEP components suggests that PEP activity requires many different components already during embryo development and that regulation of plastid transcription is complex and sophisticated from the very early stages of plant development.

PRIN2 and CSP41b were both identified in nucleoid preparations (Majeran et al., 2012) and our results suggest that these two components and possibly the PRIN2-CSP41b complex are essential for PEP dependent transcription during early embryo development. Using three independent methods CSP41b and PRIN2 were shown to directly interact (**Figure 1**). PRIN2 have two conserved Cys residues that possibly are responsible for monomer/dimer/oligomer formation upon oxidation. Consistent with this hypothesis is the observation of two bands of 20 and 40 kDa that might correspond to PRIN2 monomer and dimer (**Figure 1C**). Interestingly, the CSP41b protein migrated as continuous multimeric complex ranging from 40 to ∼700 kDa on the BN-PAGE. The presence of high molecular weight complexes was previously described for the native CSP41b protein in chloroplasts (Qi et al., 2012). Moreover, the CSP41b protein contains redox active Cys residues suggested to be putative targets of TRX (Stroher and Dietz, 2008) and the formation of high molecular weight complexes were also shown to be enhanced by oxidized conditions in the chloroplast stroma (Qi et al., 2012). When PRIN2 and CSP41b were incubated together they migrated on the gel as a distinct band (∼66 kDa; **Figure 1B**) suggesting the formation of a defined heteromeric protein complex containing both proteins. PRIN2 and CSP41b were shown to independently bind to promoter fragments of *psaA* and *psbA* in EMSA assays. However, when PRIN2 and CSP41b were incubated together, a new band of intermediate size was observed (**Figure 1D**, Supplementary data Figure S1), suggesting an interaction between PRIN2 and CSP41b also upon DNA binding *in vitro*. CSP41b was previously shown in RIP-chip analysis to be an RNA binding protein with specificity toward photosynthesis-related transcripts mainly expressed by PEP (Qi et al., 2012). However, many RNA binding proteins have also been described to have DNA binding properties, including TFIIIA, p53, STAT1, β/β′ subunits of RNAP and σ70, factors well known to regulate transcription (Sakonju et al., 1980; Clemens et al., 1993; Cassiday and Maher, 2002; Suswam et al., 2005). Another plant specific protein that binds both DNA and RNA is GUN1, a key component in retrograde communication between chloroplasts and nucleus (Koussevitzky et al., 2007). Possibly, the observed DNA binding of the PRIN2-CSP41b protein complex is required for full PEP activity as indicated by the embryo lethality of the *csp41b-2prin2.2* double mutant (**Figure 3A**).

Correlated with the embryo phenotype observed in *prin2.2* and *csp41b-2* single mutants was the impaired expression of chloroplast photosynthesis genes. The expression levels of *psaA, psbA,* and *psbD* were significantly lower in both mutants compared to wild type in MG embryos (**Figure 5C**), suggesting that the previously described defect in PEP-mediated gene expression in rosette plants is maintained in the mutants also during embryo development. Thus, our results establish a link between PEP activity and embryo development. Previously compromised translational and post-translational activities in the chloroplasts, such as mutations in elongation factor G and PPR proteins, have been shown to lead to embryo lethality (Ruppel and Hangarter, 2007; Khrouchtchova et al., 2012; Sosso et al., 2012). Interestingly, in tobacco and barley neither of the knockouts completely lacking PEP activity exhibits an embryo lethal phenotype (Allison et al., 1996; De Santis-MacIossek et al., 1999; Zhelyazkova et al., 2012). However, it should be emphasized that an essential role of plastid activity and embryo greening during embryogenesis has so far only been reported for oil-seed plants such as *Arabidopsis* and *Brassica* (He and Wu, 2009; Hsu et al., 2010). In *Arabidopsis*, PEP activity appears essential during embryo development and during this process NEP is unable to compensate for the impaired PEP activity. Recently maize mutants lacking several PEP-associated proteins such as PTACs and PRIN2 demonstrated deficiency of numerous plastid tRNAs. Thus, a role for PEP, and for the PEP associated proteins, was demonstrated for the expression of plastid transfer RNA (Williams-Carrier et al., 2014). These results emphasize the complex division of labor between NEP and PEP during the initiation of chloroplast development and that PEP, and its associated proteins, are also essential for the translation of the photosynthetic components.

## AUTHOR CONTRIBUTIONS

Dmitry Kremnev carried out the experiments and analyzed the data. Dmitry Kremnev and Åsa Strand planned the study and wrote the manuscript. Both authors read and approved the final manuscript.

## Conflict of Interest Statement

The authors declare that the research was conducted in the absence of any commercial or financial relationships that could be construed as a potential conflict of interest.
