# Supplementary material for: Plastid encoded RNA polymerase activity and expression of photosynthesis genes required for embryo and seed development in Arabidopsis
Source: Front Plant Sci. 2014 Aug 12;5:385. doi: 10.3389/fpls.2014.00385 (PMC4130184; doi:10.3389/fpls.2014.00385)
Supplement: Supplementary file 1 [file Presentation_1.PDF]

Supplementary Figure S1

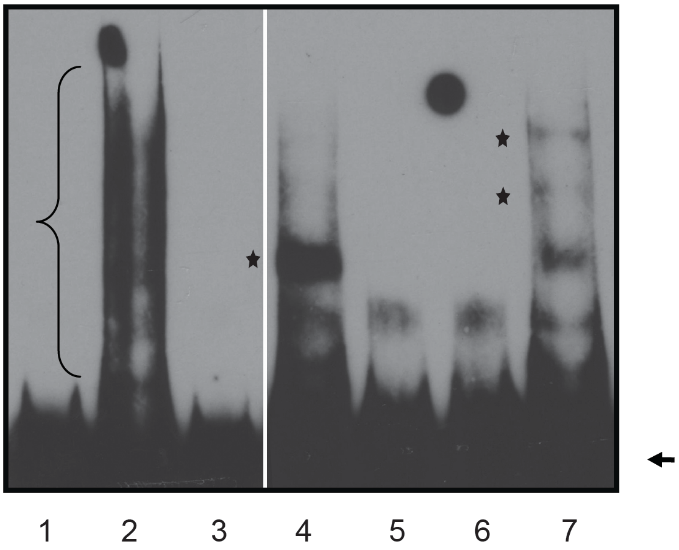

Supplementary Figure S2

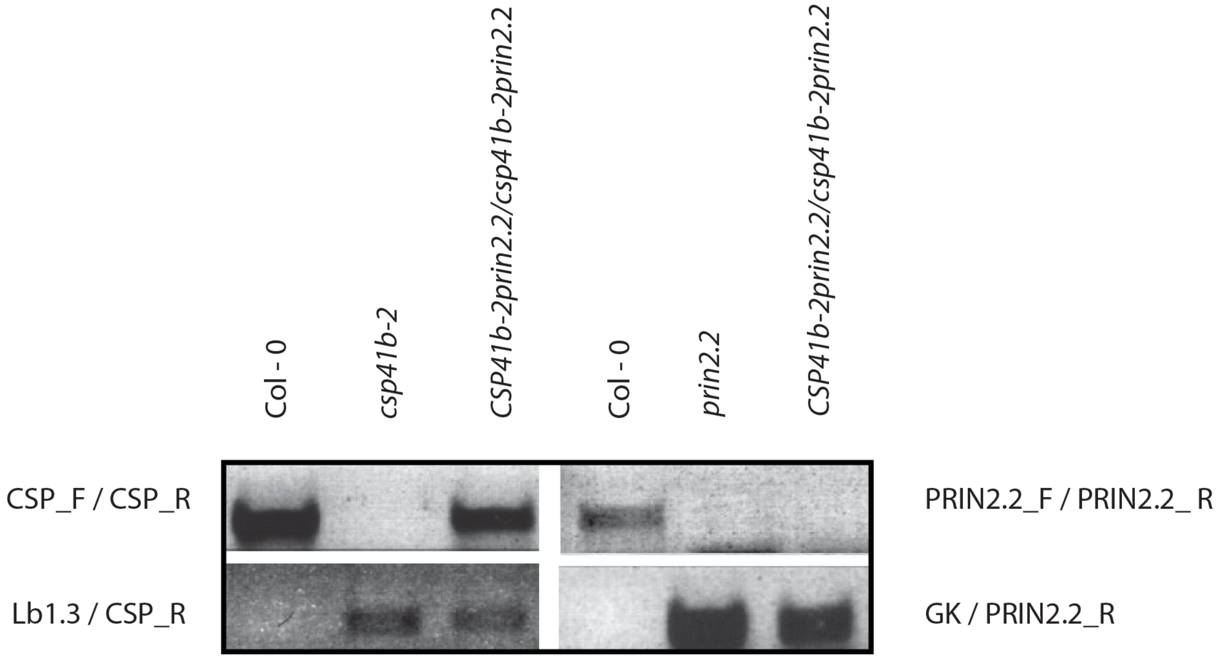

Supplementary Figure S3

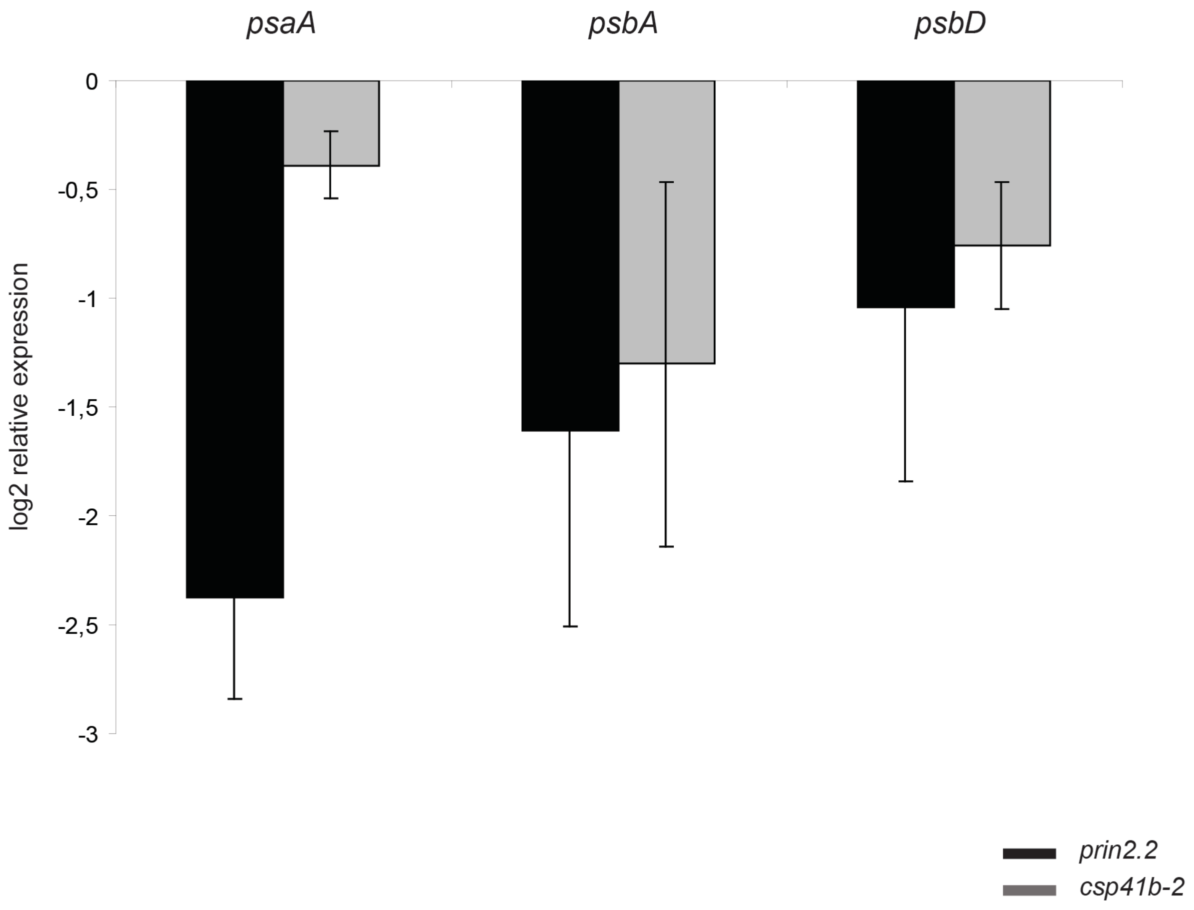

Figure legends supplementary data

FIGURE S1

***PsbA*-198bp probe binding of PRIN2 and CSP41b and their heteromerization in EMSA assay.**

Signal from *psbA*-198bp biotin labeled probe was detected by chemoluminescence nucleic acid detection module. 3µg of each purified protein was used in every reaction, DNA/protein molar ratio was 1:100. DNA/protein complexes are marked with asterisks, free DNA probe with an arrow. Competition was done with unlabeled *psbA*-198bp probe with 50 fold excess over the labeled probe. 1- free probe, 2 – probe + PRIN2, 3 - probe + PRIN2 + unlabeled probe, 4 - probe + CSP41b, 5 – probe + CSP41b + unlabeled probe, 6 - probe + PRIN2 + CSP41b + unlabeled probe, 7 - probe + PRIN2 + CSP41b.

FIGURE S2

Genotyping of *CSP41b-2prin2.2/csp41b-2prin2.2* double mutant.

Genomic DNA was extracted from leaves of 3 weeks old plants. PCR reaction with 35 cycles was set up with annealing temperature at 55°C. Combinations of Lb1.3/CSP_R and GK/PRIN2.2_R were used to detect T-DNA insertion in *CSP41b* and *PRIN2* genes respectively.

FIGURE S3

Expression levels of *psaA, psbA* and *psbD* in *prin2.2* and *csp41b-2* mature green embryos compared to the respective embryos of Col-0. Relative expression was calculated using *RCE1* (*At4g36800*) as a reference gene. Data represent means from three independent biological replicates.

Supplementary Information Kremnev and Strand

Supplementary Table 1. LC-MS/MS data of CSP41b peptides from Co-IP samples identified in two independent experiments.

Accession (Uniprot)	Protein description (Uniprot)	MW [kDa]	Similar Proteins in Uniprot	Mascot Score	Peptides matched	Sequence coverage [%]
Y1934_ARATH	Uncharacterized protein At1g09340, chloroplastic OS=Arabidopsis thaliana GN=At1g09340 PE=1 SV=1	42.6	1	327,1	7	19.0
Y1934_ARATH	Uncharacterized protein At1g09340, chloroplastic OS=Arabidopsis thaliana GN=At1g09340 PE=1 SV=1	42.6	1	278,5	6 (6)	18.3

Supplementary Table 2. Sequence information for primers used in this manuscript

Primer name	Sequence
**Protein expression**	
PRIN2_F	GCTTCCATGGGCTCCCGGAGAGGGTTCGTT
PRIN2_R	GCTTGGTACCCTAATCAGTGCCGGTCCAT
CSP41b_F	GCTTCCATGGCGAAGATGATGATGTTGCAA
CSP41b_R	GCTTGGTACCTTATTGAAGAACAAGTTTCTT
**EMSA**	
psaA_197 F	ATGACATATCCATAGGGTGCTC
psaA_197 R	TAATGACTAATAAGCATTTATT
psbA_197 F	GAAACAGTATAACATGACTTAT
psbA_197 R	ACCCACTACGGATCGTATTCAA
**Real-time primers**	
RCE1 F	CTGTTCACGGAACCCAATTC
RCE1 R	GGAAAAAGGTCTGACCGACA
PP2AA3_F	TAACGTGGCCAAAATGATGC
PP2AA3_R	GTTCTCCACAACCGCTTGGT
psaA_F	ACTACCACTTGGATCTGGAAC
psaA_R	AAACGAGCACCGTGGAAATAC
psbA_F	ATACAACGGCGGTCCTTATG
psbA_R	AGCAATCCAAGGACGCATAC
psbD_F	TCATGGTATACTCATGGATTGG
psbD_R	GACCACCTAATTGACACCAACG
rbcL_F	GTGTTGGGTTCAAAGCTGGT
rbcL_R	GTGGAACTCCAGGTTGAGGA
rpoB_F	CGAATAGCCCCTTTTGATGA
rpoB_R	CCTGGATACTCGGGTTCAAA
accD_F	AAGCGGAAAGATTCGTGAAA

accD_R	TGTCGCATTGAATCCACAAT
ycf2_F	GGTACTTCGAGCTTGGAACG
ycf2_R	TTCATCGGGTACGGGTAGAG
